# Heart Rate Variability in Sleeping Preterm Neonates Exposed to Cool and Warm Thermal Conditions

**DOI:** 10.1371/journal.pone.0068211

**Published:** 2013-07-01

**Authors:** Erwan Stéphan-Blanchard, Karen Chardon, André Léké, Stéphane Delanaud, Véronique Bach, Frédéric Telliez

**Affiliations:** 1 PériTox-INERIS Laboratory, Jules Verne University of Picardy, Amiens, France; 2 Neonatal and Pediatric Intensive Care Unit, Amiens University Medical Center, Amiens, France; University of Adelaide, Australia

## Abstract

Sudden infant death syndrome (SIDS) remains the main cause of postneonatal infant death. Thermal stress is a major risk factor and makes infants more vulnerable to SIDS. Although it has been suggested that thermal stress could lead to SIDS by disrupting autonomic functions, clinical and physiopathological data on this hypothesis are scarce. We evaluated the influence of ambient temperature on autonomic nervous activity during sleep in thirty-four preterm neonates (mean ± SD gestational age: 31.4±1.5 weeks, postmenstrual age: 36.2±0.9 weeks). Heart rate variability was assessed as a function of the sleep stage at three different ambient temperatures (thermoneutrality and warm and cool thermal conditions). An elevated ambient temperature was associated with a higher basal heart rate and lower short- and long-term variability in all sleep stages, together with higher sympathetic activity and lower parasympathetic activity. Our study results showed that modification of the ambient temperature led to significant changes in autonomic nervous system control in sleeping preterm neonates. The latter changes are very similar to those observed in infants at risk of SIDS. Our findings may provide greater insight into the thermally-induced disease mechanisms related to SIDS and may help improve prevention strategies.

## Introduction

Autonomic dysfunction has long been incriminated as a possible cause of sudden infant death syndrome (SIDS). When compared with control infants, infants who subsequently died from SIDS were seen to have a higher baseline heart rate (HR) [1], lower overall heart rate variability (HRV) [2], [3], prolonged QT indexes [4], lower parasympathetic activity and/or higher sympathovagal balance [5], [6].

It has been suggested that the interaction between sleep and thermoregulatory processes is one of the main factors associated with autonomic dysfunction [7]. Non-thermoneutral environmental temperatures increase an infant's vulnerability to SIDS [8]–[10]. Strikingly, very few data are available concerning the association between the ambient temperature and autonomic nervous activity during sleep. Indeed, the scientific literature provides an incomplete picture of the possible risks associated with non-thermoneutral ambient temperatures [11], [12].

We therefore reasoned that studying neonatal HRV might provide relevant information on (i) the interaction between sleep processes and thermoregulation and (ii) potential dysfunction of autonomic nervous control related to SIDS [13]. To this end, we investigated the effects of warm and cool thermal conditions on HRV in healthy preterm neonates as a function of the sleep stages and considered the implications of our results for SIDS prevention.

## Methods

### Patients

The study was conducted between October 2006 and May 2009 in the Neonatal Intensive Care Unit at Amiens University Medical Center (Amiens, France). Thirty-four healthy preterm neonates (mean ± s.d. gestational age: 31.4±1.5 weeks, birth weight: 1379±349 g) were recruited near-term (postmenstrual age: 36.2±0.9 weeks, weight at the time of the study: 2083±277 g) after the parents had given their written, informed consent. The protocol was approved by the local investigational review board (“Comité Consultatif de Protection des Personnes dans la Recherche Biomédicale de Picardie”, project n°99H45a1,a2,a – 97H11). All neonates were free of neurologic, respiratory and cardiac disorders and had not received treatments that could have modified their ventilatory and cardiac parameters (such as cardiotonic drugs). Although some infants had received mechanical ventilation, all those studied here had been free of ventilatory support for at least the 7 days preceding the study.

### Study design

Monitoring took place in a quiet, darkened room, with a constant air temperature of 22–24°C. Study recording began after a feed at 08:00 and continued until the neonate awoke for an 11:00 feed, so that each neonate had at least 150 minutes of uninterrupted sleep data. The neonate (wearing a diaper only) was placed in the supine position in a closed, convectively heated incubator (Médipréma ISIS®, Chambray-les-Tours, France). The incubator air temperature was adjusted using a servo-controlled, skin temperature time-derivative heating system that allows the air temperature to reach a level corresponding to individual thermoneutrality for each neonate [14]. Hence, the incubator temperature at equilibrium corresponds to an optimal thermal environment in which temperature-induced alterations in the heart and ventilation rates are avoided. The air humidity was 1.01–1.03 kPa and the air velocity was 0.01 m.s^−1^, which correspond to natural convection conditions.

### Data acquisition

Electrophysiological recordings included two electro-encephalograms (EEGs, measured from the right and the left centro-occipital leads) and eye movements (monitored by a piezoelectric quartz transducer attached to an eyelid). An electrocardiogram (ECG) was recorded using patch electrodes placed in a lead II position on the chest wall. The respiratory signal was derived from the ECG electrodes' transthoracic impedance signals. Body movements were recorded by accelerometers attached to a wrist and the opposite ankle. Transcutaneous arterial oxygen saturation values were recorded with a pulse oximeter (Oximax MAX-N, Tyco Healthcare group LP, Nellcor Puritan Bennett Division, CA). The incubator air temperature (T_inc_, °C) was measured with a thermocouple (accuracy ±0.10°C; model K; Bioblock®, Illkirch, France) located 10 cm above the infant's head. The mean skin temperature (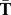
_sk_, °C) was calculated by averaging abdominal and cheek skin temperatures measured by thermocouples. Rectal temperature (T_rec_, °C) was measured with a thermistor probe (accuracy ±0.10°C; YSI 402; Bioblock®, Illkirch, France) located 1 cm inside the infant's rectum. All recordings were continuously monitored on a polysomnograph (Alice 4, Respironics®, Nantes, France).

### Procedure

For each neonate, three experimental temperature conditions were applied separately during three successive morning naps. On the first morning, T_inc_ was set to thermoneutrality (T_N_) according to the servo-controlled, skin temperature time-derivative heating system described above. The average equilibrium temperature (T_inc_: 32.49±1°C) corresponded to an optimal thermal environment that was close to (i) the thermoneutral air temperature recommended by Sauer et al. [15] and (ii) the values defined by Hey and Katz [16] in terms of birth weight and postnatal age. The warm condition (on the second morning) and the cool condition (on the third morning) corresponded to T_N_+2°C and T_N_-2°C, respectively. Hence, the warm and cool exposures were set as a function of each neonate's thermoregulatory requirements. The experimental sequence (successively thermoneutrality, followed by the warm condition and then the cool condition) was chosen in order to rule out thermal adaptation to cool exposure, since the latter can modify thermoregulatory responses and sleep structure [17], [18]. Exposure to the warm environment (T_inc_: 34.13±0.73°C) was associated with significantly higher T_rec_ (+0.23°C; *P* = 0.001) and 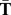
_sk_ (+0.26°C; *P*<0.001) values, when compared with thermoneutrality. Exposure to the cool incubator temperature (T_inc_: 30.38±0.68°C) was associated with slightly lower T_rec_ (−0.08°C) and 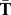
_sk_ (−0.08°C) values, relative to thermoneutrality. However, these differences were not statistically significant (Figure S1).

### Sleep scoring

Sleep stages were scored visually in 30-second periods, as recommended by the Pediatric Task Force [19]. Sleep stages were differentiated on the basis of concordance between the EEG and rapid eye movement (REM) signals. Wakefulness was defined as moments when the infant's eyes were open (whether scanning the environment or not) and when frequent body movements occurred. Crying or fussing was sometimes observed. Active sleep (AS) was defined as continuous EEG activity with REM, while quiet sleep (QS) was defined as discontinuous EEG activity in the absence of REM. Periods that did not fulfill the criteria for either AS or QS (i.e. discontinuous EEG with REM or continuous EEG in the absence of REM) were defined as indeterminate sleep. All artifactual or other events that might have influenced the infant's HR (such as a deep breath, apnea or movement) were identified manually and excluded from the analysis.

### Heart rate variability analysis

Digitized ECG signals were sampled at 2000 Hz. The intervals between the successive R waves were measured with Kubios HRV® signal analysis software (Biosignal Analysis and Medical Imaging Group, Department of Physics, University of Kuopio, Kuopio, Finland). The resulting R-points were visually inspected to ensure that the records did not contain any ectopic beats or artifacts (i.e. the RR tachogram contained only ‘normal’ beats (NN intervals)). Non-stationary trends were removed from the HRV signals to the greatest extent possible by using a detrending procedure based on smoothness priors regularization.

### Time-domain HRV parameters

The NN intervals were used to calculate four standard time-domain HRV parameters: the mean HR (bpm), the standard deviation of the NN interval (SDNN, ms, which reflects all the cyclic components responsible for variability during the recording period as variance is mathematically equal to total power of spectral analysis); the square root of the mean-squared differences of successive NN intervals (r-MSSD, ms, which is a measure of short-term variability) and the percentage of successive NN intervals that differ by more than 25 ms (pNN25, %).

These time-domain HRV parameters have been shown to be sensitive to various pharmacological interventions. During sleep, HRV is primarily determined by vagal activity and an increased variability reflects a higher parasympathetic modulation of the sinus node. Accordingly, SDNN and r-MSSD were consistently found to increase β-adrenergic receptor blockade [20] and to decrease after parasympathetic blockade [21].

### Frequency-domain HRV parameters

Power spectra were computed with a fast Fourier transform based on Welch's averaged periodogram method [22]. Prior to estimation of the power spectral density, the NN interval series was converted to equidistantly sampled series by applying a cubic spline interpolation method. For each subject, the HRV power spectra were generated from artifact-free, stationary 2-min recording segments selected by visual inspection. The spectral analysis focused on three main bandwidths: the very low-frequency band (VLF, 0.003–0.04 Hz), the low-frequency band (LF, 0.04–0.24 Hz) and the high-frequency band (HF, 0.24–1.04 Hz). The upper limit of HF variability components was set at a higher value than usual, in order to take into account the high respiratory rates observed in infants [23], [24]. The power in each of the three bands was extracted and expressed an absolute value (ms^2^) and a relative value (%). Sympathovagal balance was expressed as the LF/HF ratio.

### Poincaré plot

The Poincaré plot is a graphical representation of the correlation between successive RR intervals, i.e. plot of RR*_n_*_+1_ as a function of RR*_n_* (Figure S2 showing a representative plot for a study subject). Heart rate variability was quantified from the Poincaré plot data by extracting the standard deviations SD1 (ms) and SD2 (ms). SD1 is the standard deviation of the change between successive RR intervals (i.e. the points perpendicular to the line of identity) and is a measure of short-term variability, whereas SD2 is the standard deviation of the beat intervals (i.e. along the line of identity) and characterizes long-term variability. Two further criteria [25] were computed from SD1 and SD2: the cardiac vagal index (CVI = log (SD1×SD2)) and the cardiac sympathetic index (CSI = SD2/SD1).

### Statistical analysis

Statistics were computed by using Statview software (SAS Institute, Inc., Cary, NC). Values expressed as percentage (pNN25, relative values of VLF, LF and HF) were arcsined transformed to stabilize the variance. The normality of the data distribution was checked with a Kolmogorov-Smirnov test. A Box-Cox transformation was used on the values that did not follow a normal distribution (absolute values of VLF, LF and HF). Two-way analyses of variance for repeated measures and paired *t* tests were used to test the effects of thermal conditions and sleep stages on the different temperatures and on HRV parameters. Since the HR was found to correlate with some HRV parameters in previous studies [26], statistical analyses were repeated including HR as a covariate. However, controlling for HR did not change the results. Since no interaction between thermal conditions and sleep stages was found, the AS and QS values were pooled for subsequent analyses. The threshold for statistical significance was set to *P*<.05. Data are quoted as the mean ± s.d.

## Results

### Sleep and ventilatory data

An influence of thermal condition was found on sleep structure (Table 1). The duration of total sleep time was significantly lower in the cool condition as compared with thermoneutrality (*P* = 0.0197) and the warm condition (*P* = 0.0023). This effect was mainly due to an increase in the duration of wakefulness after sleep onset, which was significantly higher in the cool condition as compared with thermoneutrality (*P* = 0.0004) and the warm condition (*P* = 0.006). Furthermore, the proportion of QS was significantly higher in the warm condition than in the cool condition (*P* = 0.0257).

**Table 1:** Sleep and ventilatory data in the cool condition (T_N_-2°C), at thermoneutrality (T_N_) and in the warm condition (T_N_+2°C).

	T_N_-2°C	T_N_	T_N_+2°C	Thermal condition effect
Total sleep time (min)	128.4±13.1	135.9±11	139.9±17.2	*P* = 0.0044
Wakefulness after sleep onset (min)	26.6±13.1	15.2±9.8	16.5±10.9	*P*<0.001
Active sleep (%)	64.5±11	61.8±9.7	60.9±11.1	NS
Quiet sleep (%)	21.8±7.1	25.4±6.5	26.1±7.8	*P* = 0.0401
Respiratory rate (breaths.min^−1^)	54.5±9.9	52.6±10.3	54.9±8.7	NS

Values are quoted as the mean ± standard deviation. NS: not significant.

A significant effect of sleep stage was observed for respiratory rate, which was higher during AS than during QS (*P*<0.001). No significant effect or interaction was found for thermal condition (Table 1).

### Time-domain HRV parameters

The mean values of HR and time-domain HRV parameters in the three thermal conditions according to AS and QS are shown in Table 2. A significant influence of sleep stage was observed for HR and SDNN, which were higher during AS than during QS. A significant influence of thermal condition was found on HR, SDNN, r-MSSD and pNN25. The heart rate was significantly higher in the warm condition than for thermoneutrality or in the cold condition (*P*<0.001). There was no significant difference between thermoneutrality and the cold condition in terms of HR. Interestingly, SDNN, r-MSSD and pNN25 were highest in the cool condition and lowest in the warm condition in both sleep stages. There was a statistically significant difference in SDNN when comparing the cool condition with thermoneutrality (*P* = 0.0066) and in r-MSSD (*P*<0.001) and pNN25 (*P* = 0.0129) when comparing the warm condition with thermoneutrality.

**Table 2:** Heart rate and time-domain HRV parameters in the cool condition (T_N_-2°C), at thermoneutrality (T_N_) and in the warm condition (T_N_+2°C) during active sleep (AS) and quiet sleep (QS).

	T_N_-2°C		T_N_		T_N_+2°C		Thermal condition Effect	Sleep stage effect
	AS	QS	AS	QS	AS	QS		
HR (bpm)	148.0±10.2	144.3±12.0	148.8±8.0	144.0±10.2	152.2±9.4	150.5±10.4	*P* = 0.0023	*P*<0.001
SDNN (ms)	26.0±10.7	20.2±8.7	20.6±6.0	15.3±7.5	18.6±6.3	15.4±8.4	*P*<0.001	*P* = 0.0025
r-MSSD (ms)	11.8±5.8	12.7±7.0	10.7±5.6	9.4±5.2	8.6±4.5	7.3±4.0	*P* = 0.005	NS
pNN25 (%)	7.2±9.5	8.7±10.3	6.7±9.3	3.9±5.7	3.4±5.4	2.3±3.5	*P* = 0.0178	NS

Values are quoted as the mean ± standard deviation. NS: non-significant in an analysis of variance with repeated measures.

### Frequency-domain HRV parameters

The mean values of the frequency-domain HRV parameters in the three thermal conditions according to AS and QS are shown in Table 3. The LF/HF ratio and the absolute and relative values of LF were significantly higher during AS than during QS, whereas the relative values of HF were higher during QS than during AS. We observed a significant influence of thermal condition on the absolute values of VLF and HF, which were highest in the cool condition and lowest in the warm condition. This influence was statistically significant for VLF when comparing the cool condition with thermoneutrality (*P* = 0.0262) and for HF when comparing the cool condition with the warm condition (*P*<0.001).

**Table 3:** Frequency-domain HRV parameters in the cool condition (T_N_-2), at thermoneutrality (T_N_) and in the warm condition (T_N_+2) during active sleep (AS) and quiet sleep (QS).

	T_N_-2°C		T_N_		T_N_+2°C		Thermal condition effect	Sleep Stage effect
	AS	QS	AS	QS	AS	QS		
VLF (ms^2^)	359.1±396.6	248.7±364.3	240.3±231.9	136.2±179.0	171.8±193.7	161.9±228.2	*P* = 0.0112	NS
LF (ms^2^)	270.7±268.7	161.3±174.3	179.2±122.1	114.1±174.6	156.8±139.4	134.8±204.1	NS	*P* = 0.0205
HF (ms^2^)	46.1±37.5	55.3±45.2	37.9±32.7	33.4±39.2	27.5±30.1	23.1±26.3	*P* = 0.0102	NS
VLF (%)	45.3±17.5	43.6±19.3	46.5±18.6	42.2±17.6	44.2±15.7	45.3±17.4	NS	NS
LF (%)	43.2±15.0	35.5±14.0	43.5±16.3	39.5±16.9	46.5±14.4	44.2±17.1	NS	*P* = 0.012
HF (%)	11.5±10.1	21.0±22.8	10.0±7.8	18.3±18.6	9.3±11.6	10.6±11.4	NS	*P* = 0.0015
LF/HF	8.4±7.6	5.1±5.2	8.7±7.2	5.2±5.4	10.7±8.2	7.6±5.9	NS	*P* = 0.0099

Values are quoted as the mean ± standard deviation. NS: non-significant in an analysis of variance with repeated measures.

### Poincaré plot data

**Figure 1:**
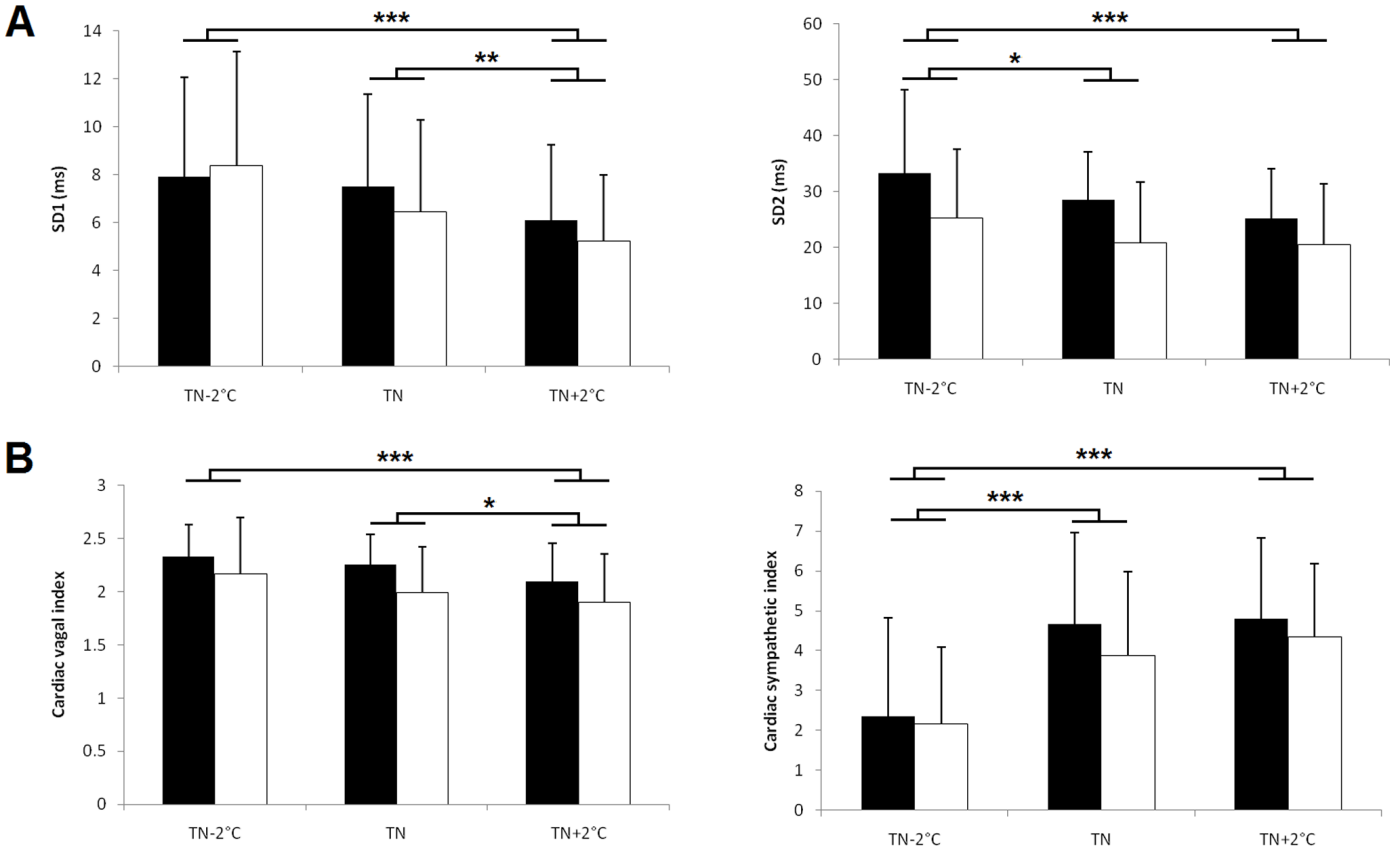
Influence of the thermal environment on HRV parameters calculated from the Poincaré plot. Mean values (± s.d.) of (A) SD1 and SD2 and (B) the cardiac vagal index and cardiac sympathetic index in the cool condition (T_N_-2°C), at thermoneutrality (T_N_) and in the warm condition (T_N_+2°C) during active sleep (empty bars) and quiet sleep (black bars). * *P*<0.05; ** *P*<0.01; *** *P*<0.001.

A significant influence of sleep stage was observed for SD2 (*P* = 0.0024) and the CVI (*P*<0.001), which were higher during AS than during QS. Furthermore, we observed a significant influence of thermal condition on SD1 (*P* = 0.0084), SD2 (*P* = 0.0019), the CVI (*P* = 0.0028) and the CSI (*P*<0.001). We showed that SD1, SD2 and the CVI were highest in the cool condition and lowest in the warm condition in both sleep stages. The opposite pattern was observed for the CSI (Figure 1). There was a significant difference between the cool condition and thermoneutrality for SD2 (*P* = 0.0271) and the CSI (*P*<0.001) and between thermoneutrality and the warm condition for SD1 (*P* = 0.0059) and the CVI (*P* = 0.0344).

## Discussion

Our study results showed that modification of the ambient temperature led to significant changes in autonomic nervous system (ANS) control in sleeping preterm neonates, independently of the sleep stage. A strength of our study lies in the demonstration that the changes in ANS activity (as evidenced by an HRV analysis) produced here by varying the ambient temperature are similar to those observed by others in SIDS victims.

### Thermal effects on autonomic nervous system activity

The servo-controlled program used in our study tailors the thermoneutral conditions in the incubator to suit each individual infant [14]. Studied infants were healthy preterm neonates and the thermal loads for cool and warm conditions (ranging from 30.4°C to 34.1°C) were within the physiological range encountered during routine care and were chosen to avoid over-complex responses. Therefore, the changes observed in the present study are thought to be normal, adaptive responses to heat stress rather than signs of dysfunctional ANS control. Under these circumstances, peripheral blood flow control is the main thermoregulatory effector in infants [27]. Previous research has suggested that an autonomic rhythm (generated by the central nervous system) is involved in vasomotor control and may be related to temperature regulation. The rhythm may regulate blood flow through skin arteriovenous anastomoses as a function of the body's thermal balance. This type of change in blood flow affects the HR and is reflected by the HRV [28], [29]. In the present study, the HR measured in the cold condition did not differ significantly from that observed at thermoneutrality but was higher in the warm condition as a result of the vasodilatory response. We also showed that a moderately elevated ambient temperature was associated with low overall HRV (as evidenced by the SDNN), as a result of decreases in both short- and long-term variability. At elevated temperatures, arterial blood pressure falls (due to peripheral vasodilatation) and the HR increases in a compensatory manner. If the temperature continues to rise, the ANS's response (i.e. an increase in sympathetic activity) reaches a ceiling and is followed by a decrease in HRV.

High HR fluctuations (i.e. short-term variability, represented here by r-MSSD, pNN25, the power spectrum in the HF band, SD1 and the CVI) reflect parasympathetic activity, which is mainly modulated by ventilatory cycles. The CVI was found to decrease significantly upon atropine-induced parasympathetic blockade [25]. In the present study, we found that an elevated environmental temperature was associated with lower short-term variability - possibly reflecting less cardiac vagal control. This finding agrees with previous data on 3-month-old term infants in whom changes in autonomic control with thermal conditions were characterized by low HF power [11], [12]. The present results also agree with studies having investigated the influence of the prone sleeping position on autonomic control in both preterm and term infants. It has been suggested that the reduction in parasympathetic control found in the prone position [30], [31] may be caused by an increase in peripheral skin temperature [32]. Our results could have been due to the interaction between thermoregulation and ventilation. Warm temperatures are known to increase respiratory rates [33]–[35], which limit respiratory sinus arrhythmia and thus may decrease the power in the HF band. However, we found that respiratory rates were not modified by thermal conditions. Hence, we cannot rule out a direct effect of thermoregulatory processes.

Long-term variability (as evidenced here by SD2, the CSI and the power spectrum in the LF and VLF bands) is characterized by slow and very slow HR fluctuations. Low-frequency changes in HR are thought to be related to baroreceptors and are primarily mediated by sympathetic activity (although parasympathetic activity also has an influence). Here, elevation of the ambient temperature also resulted in lower long-term variability parameters (with the exception of the CSI). The latter index falls significantly upon propranolol-induced sympathetic blockade [25]. The relative increase when considering cool, thermoneutral and then warm conditions suggests an increase in cardiac sympathetic control, although the power spectrum in the LF band was lower at an elevated ambient temperature. This may be due to the fact that the HR fluctuations within the LF range depend on both sympathetic and parasympathetic controls. This would imply that the LF band reflects overall HRV to a greater extent than cardiac sympathetic control does, whereas the CSI is more sensitive to changes in the sympathovagal balance. This hypothesis is supported by the fact that the LF/HF ratio was also lowest in the cool environment and highest in the warm environment (although the differences were not statistically significant). The physiological explanation of the VLF component is less well described. Thermoregulation and the renin-angiotensin-aldosterone system are the main mechanisms that have been proposed to explain these very slow HR variations. Many studies in adults have provided indirect evidence that very low frequency power may reflect (at least in part) thermoregulation to ambient temperature changes [36], [37]. In contrast, other studies failed to find the baroreflex linkage between thermoregulatory skin blood flow, arterial pressure and baroreflex-mediated HR fluctuations [38], which is a critical element of the thermoregulatory hypothesis. Furthermore, it has also been suggested that very-low-frequency HR oscillations are very much dependent on parasympathetic tone [38]. Therefore, it is not surprising that we found, in the present study, a concomitant decrease of the power in the VLF and HF bands with increasing temperature. Lastly, these observations show that an elevated ambient temperature is associated with lower overall and long-term HRV, and greater sympathetic activity.

These results should however be interpreted with caution. The increase (mean HR and the CSI) or the decrease (SDNN, r-MSSD, pNN25, the power spectrum in the VLF and HF bands, SD1, SD2 and the CVI) in significant HRV parameters with elevation of temperature did not always follow a continuous and linear progression. Differences were sometimes found between the cool condition and thermoneutrality only, or between thermoneutrality and the warm condition only. The lack of significance in some comparisons may be explained, at least in part, by a low statistical power. Physiological measures such as HRV are subject to high inter-individual differences, especially in preterm neonates. There was also a large discrepancy in the response to thermal challenges between neonates. Thus, examining individual trajectories in response to thermal conditions would be interesting. Actually, 67.7% of the neonates for the mean HR and 47.1% for the CSI showed a global increase between the cool and warm conditions. However, a continuous increase from the cool condition to thermoneutrality and then to the warm condition was found in only 40.6% of the neonates for the mean HR and 17.6% for the CSI. In the same manner, 73.5% of the neonates showed a global decrease between the cool and warm conditions (mean percentage for SDNN, r-MMSD, pNN25, the power spectrum in the VLF and HF bands, SD1, SD2 and the CVI). However, a continuous decrease from the cool condition to thermoneutrality and then to the warm condition was observed in only 31.7% of the neonates (mean percentage for the latter HRV parameters).

### Implications for SIDS

Many epidemiological studies have identified common factors in SIDS victims, such as the prone sleeping position, sleep deprivation and a number of situations related to thermal stress [39]–[41]. In particular, impairments of the organism's thermal regulation have long been linked to SIDS through known risk factors, such as head-covering, excessive insulation and an elevated room temperature [42]. It has been suggested that thermal stress could lead to death by disrupting the central control mechanisms involved in respiratory drive, the laryngeal closure reflex and/or the depression of arousal mechanisms during sleep.

We showed here that preterm infants exposed to a warm ambient temperature had a higher basal HR and lower overall HRV in all sleep stages, when compared with thermoneutrality. An elevated ambient temperature was associated with lower short- and long-term HRV, higher sympathetic activity and lower parasympathetic activity. Our results agree with previous studies in which a higher ambient temperature was associated with a higher basal HR and lower HRV [11], [12], [43], [44], and a cool environment was linked to greater autonomic dysfunction in infants at risk of SIDS [13]. Interestingly, the temperature-related changes in HRV observed in the present study are very similar to those reported in studies of the putative link between HRV and SIDS. Researchers have evidenced lower time-domain indices in all sleep stages in infants with aborted SIDS episodes [45], lower long-term variability during QS in a future SIDS victim [46] and lower standard deviation [3] in awake future SIDS victims. In studies measuring frequency-domain indices, SIDS victims and near-victims variously showed either lower parasympathetic activity in all sleep stages [5] and increased sympathetic activity during QS [47], relative to non-victims. Studies that failed to find differences between SIDS victims and controls did not differentiate between behavioral states, which may have been a confounding factor [48], [49].

## Conclusion

Our study results show clearly that the imposition of a non-thermoneutral environment is associated with changes in ANS control in sleeping preterm neonates. Examining the adaptive responses of neonates exposed to non-thermoneutral environment can lead to a better understanding of potential impairments, better prediction of outcomes and, potentially, more effective compensatory interventions in the clinic. This study may pave the way for further investigations of the putative relationship between the changes in ANS activity and the life-threatening events implicated in SIDS. There is also a need for prospective studies of the value of autonomic function tests for predicting the SIDS risk in infants and the effect of associated epidemiological factors. Lastly, it is essential to continue public health efforts which aimed at decreasing known environmental risk factors.

## Supporting Information

Figure S1 **Mean (± s.d.) incubator, rectal and skin temperatures in the cool condition (empty bars), at thermoneutrality (gray bars) and in the warm condition (black bars).** * *P*<0.05; ** *P*<0.01; *** *P*<0.001.
(TIF)

Figure S2 **Example of a Poincaré plot (a graphical representation of RR** ***_n_***_**+1**_ **as a function of RR** ***_n_*** **) using data from a subject during quiet sleep.** The HRV parameters SD1 (standard deviation of the change between successive RR intervals, i.e. the points perpendicular to the line of identity) and SD2 (standard deviation of the beat intervals, i.e. along the line of identity) were calculated from the plot.
(TIF)
