# Supplementary figures and images for: Heart Rate Variability in Sleeping Preterm Neonates Exposed to Cool and Warm Thermal Conditions
Source: PLoS One. 2013 Jul 1;8(7):e68211. doi: 10.1371/journal.pone.0068211 (PMC3698119; doi:10.1371/journal.pone.0068211)

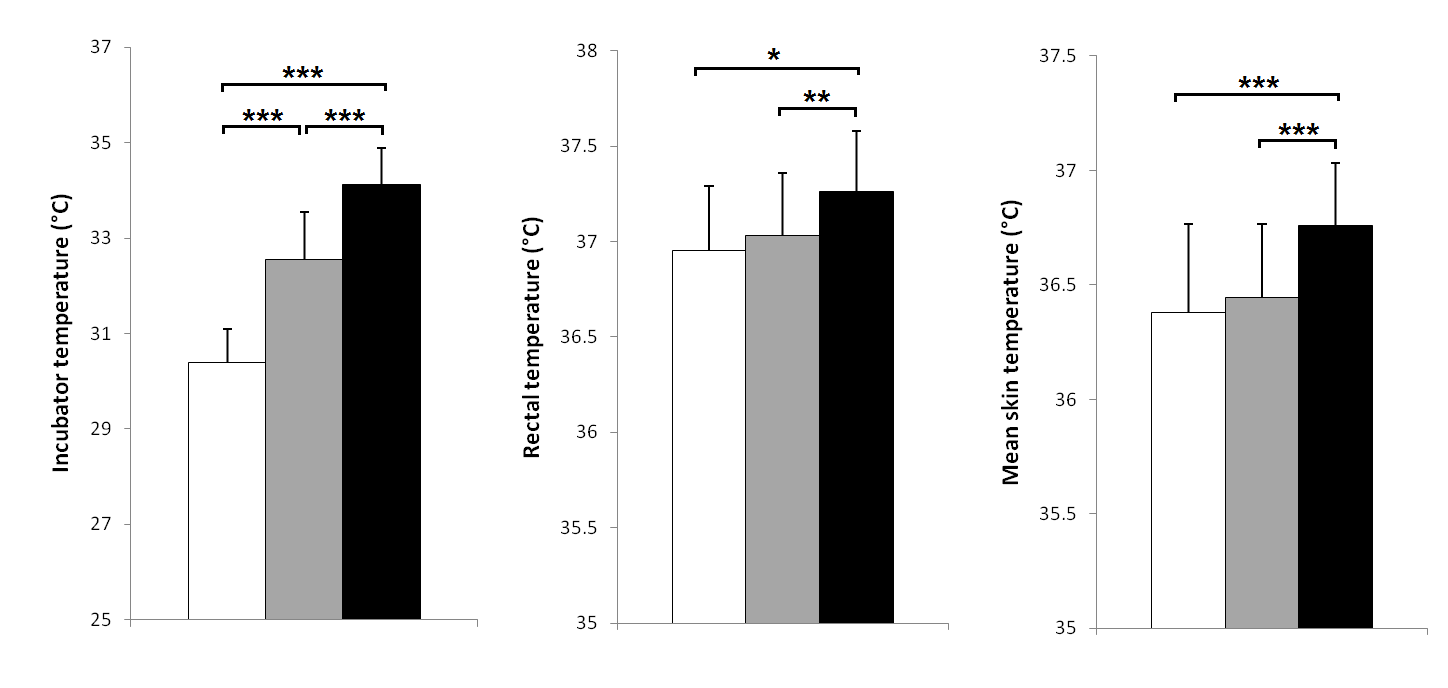

Supplement: Figure S1 — Mean (± s.d.) incubator, rectal and skin temperatures in the cool condition (empty bars), at thermoneutrality (gray bars) and in the warm condition (black bars). * P<0.05; ** P<0.01; *** P<0.001. (TIF) [file pone.0068211.s001.tif]

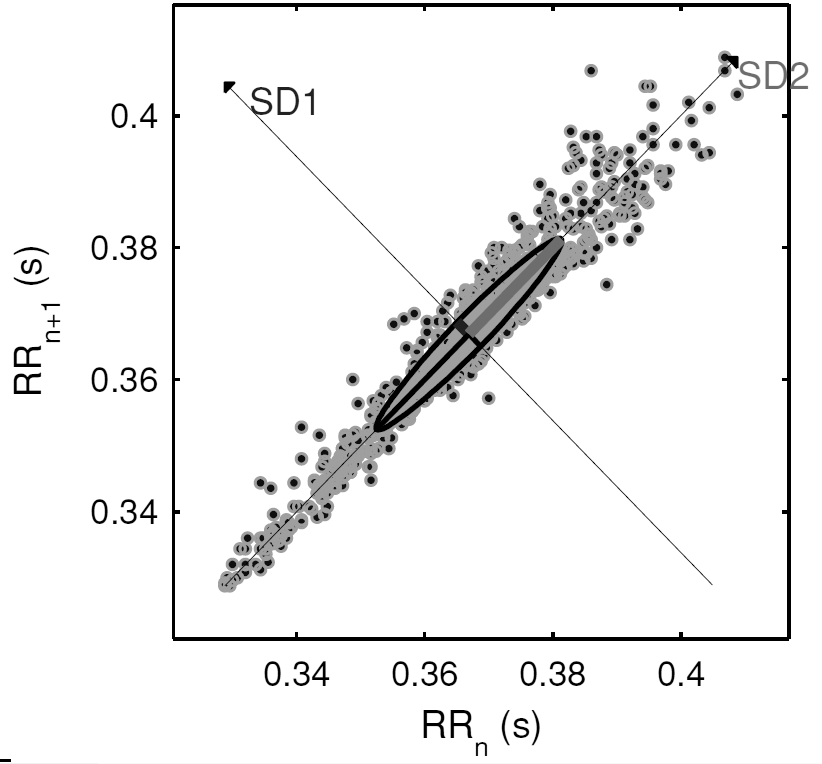

Supplement: Figure S2 — Example of a Poincaré plot (a graphical representation of RR n +1 as a function of RR n ) using data from a subject during quiet sleep. The HRV parameters SD1 (standard deviation of the change between successive RR intervals, i.e. the points perpendicular to the line of identity) and SD2 (standard deviation of the beat intervals, i.e. along the line of identity) were calculated from the plot. (TIF) [file pone.0068211.s002.tif]
